# Effects of Dietary Host-Derived *Bacillus*–Fructo-Oligosaccharide Formulations on Growth Performance and Thermal Challenge Responses in Juvenile Olive Flounder (*Paralichthys olivaceus*)

**DOI:** 10.3390/ani16111655

**Published:** 2026-05-28

**Authors:** Hyuncheol Jeon, Haham Kim, Sooa Yoon, Suhyun Lee, Md Hashibur Rahman, Sungchul C. Bai, Su-Jeong Lee, Eun-Woo Lee, Taesun Min, Mohammad Moniruzzaman, Seunghyung Lee

**Affiliations:** 1 Interdisciplinary Program of Blue Food, Pukyong National University, Busan 48513, Republic of Korea; conjp@naver.com; 2 Major of Aquaculture and Applied Life Sciences, Division of Fisheries Life Sciences, Pukyong National University, Busan 48547, Republic of Korea; haham7@naver.com (H.K.); dbshelena@naver.com (S.Y.); su8842@naver.com (S.L.); hashibur@pukyong.ac.kr (M.H.R.); scbai@pknu.ac.kr (S.C.B.); 3 Interdisciplinary Program of Marine and Fisheries Sciences and Convergent Technology, Pukyong National University, Busan 48513, Republic of Korea; 4 Feeds and Foods Nutrition Research Center, Pukyong National University, Busan 48547, Republic of Korea; 5 Department of Biopharmaceutics, Division of Applied Bioengineering, Dong-Eui University, Busan 47340, Republic of Korea; xqil1on@gmail.com (S.-J.L.); ewlee@deu.ac.kr (E.-W.L.); 6 Department of Animal Biotechnology, Jeju International Animal Research Center, Sustainable Agriculture Research Institute (SARI), Jeju National University, Jeju 63243, Republic of Korea; tsmin@jejunu.ac.kr

**Keywords:** climate change, environmental stressor, thermal tolerance, synbiotics, *Bacillus* spp.

## Abstract

This study evaluated the effects of dietary host-derived *Bacillus* strains combined with fructo-oligosaccharide (FOS) on growth performance, basal physiological status, intestinal morphology, and thermal stress responses in juvenile olive flounder (*Paralichthys olivaceus*). A total of 486 fish with an initial body weight of 7.26 ± 0.04 g were randomly distributed into 27 tanks and fed nine experimental diets for nine weeks. The diets consisted of a basal control, an FOS-only diet, three single-strain synbiotic diets containing FOS and one host-derived *Bacillus* strain (*B. sonorensis*, *B. subtilis*, or *B. velezensis*), and four multi-strain synbiotic diets containing FOS and combinations of two or three strains. Probiotics were included at 1 × 10^7^ CFU g^−1^ diet, and FOS was supplemented at 5 g kg^−1^ diet. After the feeding trial, no significant dietary effects were observed on growth performance, somatic indices, whole-body proximate composition, plasma biochemical parameters, antioxidant enzyme activities, immune-related indicators, stress-related biomarkers, or intestinal morphology. Fish were subsequently subjected to lethal and acute high-temperature challenges to evaluate thermal stress tolerance and associated physiological responses. In the lethal temperature challenge, fish fed the multi-strain diets FOS + *B. sonorensis* + *B. velezensis* and FOS + *B. sonorensis* + *B. subtilis* + *B. velezensis* showed numerically higher survival than the other groups; however, these differences were not statistically significant. Following acute heat exposure, dietary treatments did not significantly affect plasma metabolites, and most heat-shock- and energy-metabolism-related genes were not differentially expressed among treatments. Hepatic *AMPKβ* expression showed a significant dietary treatment effect, with higher expression in the BCF and ACF groups than in the ABF group. Overall, host-derived synbiotic supplementation did not significantly enhance growth performance or basal physiological responses under the present experimental conditions. However, some multi-strain combinations showed a non-significant tendency toward higher survival under lethal thermal stress. Further studies incorporating gut microbiome profiling, metabolomic analysis, and alternative dietary conditions are required to clarify whether host-derived *Bacillus*–FOS synbiotic formulations can influence thermal stress resilience in olive flounder.

## Simple Summary

Climate-driven increases in summer water temperature are an important stress factor influencing the survival of olive flounder in aquaculture, an important farmed marine fish in East Asia. To improve stress resistance, synbiotics may be used as a dietary tool, as they combine beneficial bacteria with growth-promoting prebiotic substrates. In this study, juvenile olive flounder were fed diets containing host-drived *Bacillus* strains, fructo-oligosaccharides, or their combinations for nine weeks to test whether these supplements could improve growth and resistance to heat stress. The growth performance was not improved by the dietary treatments. However, fish fed some multi-strain synbiotic diets tended to survive a lethal heat challenge better than fish fed the other diets, although the difference was not statistically significant. No distinct dietary effects were observed on plasma indicators or the relative expression of heat-shock- and energy-metabolism-related genes after short-term thermal stress.

## 1. Introduction

Olive flounder (*Paralichthys olivaceus*) is the most commercially significant marine finfish species in Korea, accounting for over 50% of the country’s total marine aquaculture production [1]. Although olive flounder is classified as a eurythermal species capable of tolerating a relatively wide temperature range [2], its aquaculture production is increasingly threatened by climate-driven thermal instability, particularly recurrent summer heatwaves and elevated seawater temperatures [3,4]. Rapid shifts in sea surface temperature and the increased frequency of heatwave events have been widely reported in coastal waters, including areas supporting olive flounder aquaculture [5]. Because olive flounder is a poikilothermic marine species with optimal performance within a relatively narrow thermal range, approximately 18–22 °C, deviations beyond this range can severely disrupt metabolic processes, feeding activity, immune competence, and physiological homeostasis [6,7]. Acute thermal fluctuations under current climate scenarios can induce oxidative stress, suppress growth, and increase mortality in this species, underscoring the biological and economic vulnerability of flounder culture [8,9]. Thermal stress also directly impairs digestive function, immune competence, metabolic efficiency, and overall health in poikilothermic organisms such as fish, making them especially vulnerable to climate-driven environmental instability [10,11]. From an industrial perspective, these thermal-stress-associated responses represent major production constraints because they can reduce growth efficiency, increase disease susceptibility and mortality risk, and compromise the stability and profitability of olive flounder farming. Therefore, nutritional and microbial feed-based strategies that support physiological homeostasis and thermal stress resilience are of practical importance for sustainable olive flounder aquaculture [12].

Probiotics are defined as microbial organisms or their functional components that provide discernible health benefits to the host when administered in adequate concentrations [13]. In aquaculture, probiotics have gained substantial attention as sustainable tools for improving nutrient utilization, immune modulation, and water quality, thereby reducing reliance on chemotherapeutic agents and enhancing system resilience [14,15]. Prebiotics are non-digestible dietary substrates that specifically promote the proliferation and metabolic functions of beneficial intestinal microorganisms [16] and may further support microbial activity through fermentation-derived metabolites associated with gut integrity and immune responses [17]. The concurrent application of probiotics and prebiotics, referred to as synbiotics, has been reported to influence survival, microbial stability, and physiological responses in fish [18,19,20]. In the context of olive flounder aquaculture, such microbial-based dietary interventions should be considered practical tools for improving host physiological robustness rather than as the central research subject itself. Their relevance lies in whether they can help olive flounder maintain metabolic, immune, and stress-related stability during thermal challenges. In the context of unprecedented climate change, marked by heightened frequency and intensity of thermal alterations and heat-stress events, such dietary approaches have gained particular significance [21].

However, although microbial-based strategies are increasingly used to improve stress resilience in aquaculture, numerous probiotics currently utilized in fish production originate from terrestrial or non-host sources [22]. These strains often exhibit limited adhesion, colonization, and persistence in the fish gastrointestinal tract, thereby potentially reducing their capacity to maintain functionality under physiologically challenging conditions, including heat stress [23,24,25]. Host-associated probiotics (HAPs) refer to microbial strains isolated from the gastrointestinal tract, mucosal surfaces, or rearing environment of the target fish species and subsequently reintroduced to enhance host nutrition, immune function, and overall resilience [24]. In contrast to probiotics originating from terrestrial sources, HAPs may offer greater ecological compatibility with the aquatic environment and the host’s physiological conditions, which could favor colonization, persistence, and functional activity within the gastrointestinal tract [26,27]. This intrinsic adaptability suggests that HAPs may be useful candidates for supporting metabolic performance, immune responses, and disease resistance in farmed fish species [28]. For olive flounder, host-associated *Bacillus* strains are particularly relevant because they originate from the target host and may therefore be better suited to interact with the intestinal environment of this species than non-host-derived strains. When combined with fructo-oligosaccharide (FOS), these strains can be formulated as host-associated *Bacillus*–FOS synbiotic formulations with potential application as functional feed additives for improving stress resilience in olive flounder aquaculture.

The microbiome is broadly defined as the collective community of microorganisms, including bacteria, archaea, fungi, viruses, and protozoa, inhabiting a particular environment [29]. In vertebrates, the gut microbiota, the microbial consortium residing in the intestinal tract, plays a central role in host physiology by contributing to nutrient digestion and absorption, vitamin synthesis, immune development, pathogen exclusion, and maintenance of mucosal barrier integrity [30,31,32]. In fish, disruption of the microbial community, known as dysbiosis, is linked to impaired intestinal epithelial function, altered nutrient utilization, metabolic imbalance, reduced immune responses, higher susceptibility to enteric pathogens, reduced growth, behavioral changes, and increased mortality [33,34]. These observations indicate that modulation of the gut microbiota is relevant not only to growth and health but also to broader aspects of fish physiology and performance. An emerging body of evidence indicates that synbiotics can influence these microbiota–host interactions [35]. The gut–brain axis is a bidirectional communication network connecting the central nervous system with the gastrointestinal tract through neural, endocrine, and immune pathways, and it is increasingly recognized as an interface through which the microbiota may influence host behavior, stress responses, and metabolic function [36]. Gut microorganisms can produce neuroactive compounds, including gamma-aminobutyric acid (GABA), dopamine, and noradrenaline, as well as short-chain fatty acids (SCFAs), which function as signaling molecules within this axis and may influence host physiology through neuroendocrine and mucosal immune pathways [37]. In fish models such as zebrafish, dietary synbiotics have been shown to modify gut microbial composition, decrease corticotropin-releasing hormone (CRH) expression, reduce circulating cortisol levels, and alleviate stress-related behavioral and physiological responses [38,39]. Although these findings provide a mechanistic rationale for using synbiotic formulations as nutritional tools, evidence directly linking host-derived *Bacillus*–FOS synbiotic formulations to thermal stress responses in olive flounder remains insufficiently characterized.

Despite the commercial importance of olive flounder and its vulnerability to high-temperature stress, several species-specific scientific questions remain unresolved. First, it is unclear whether host-associated *Bacillus* strains combined with FOS can improve growth performance and physiological condition under normal rearing conditions in juvenile olive flounder. Second, it remains uncertain whether single-strain and multi-strain synbiotic formulations differ in their capacity to support thermal stress resilience. Third, limited information is available on whether these dietary interventions can influence host-level stress responses, including plasma biochemical changes, antioxidant and immune-related parameters, intestinal morphology, survival under lethal temperature exposure, and heat-shock- or energy-metabolism-related gene expression after acute thermal challenge. Most previous studies on probiotics and synbiotics in aquaculture have focused on growth, immunity, or pathogen resistance under optimal environmental conditions, whereas their application to olive flounder under acute or lethal thermal stress has not been sufficiently evaluated. Moreover, the potential of host-derived *Bacillus* strains in this context requires further investigation, despite their ecological relevance and presumed compatibility with the host intestinal environment compared with non-host probiotics.

Based on this background, we hypothesized that dietary supplementation with host-associated *Bacillus*–FOS synbiotic formulations, particularly multi-strain formulations, would improve physiological status under normal rearing conditions and enhance thermal stress resilience in juvenile olive flounder. Specifically, we expected these dietary treatments to improve growth-related performance and host physiological indicators during the feeding period and to be associated with improved survival, altered plasma metabolites, and modulation of heat-shock- and energy-metabolism-related gene expression following thermal challenge. To test this hypothesis, the current study evaluated the effects of dietary supplementation with host-associated *Bacillus* strains and FOS on growth performance, thermal stress responses, and plasma biochemical responses in juvenile olive flounder. The study included growth performance, whole-body composition, plasma-related parameters, antioxidant enzyme activities, immune-related indicators, stress-related biomarkers, intestinal morphology, lethal-temperature survival, and gene expression responses after acute heat exposure. By integrating growth performance metrics with physiological, histological, molecular, and survival outcomes, this study aimed to provide a species-specific assessment of whether host-associated *Bacillus*–FOS synbiotic formulations are associated with measurable changes in the capacity of olive flounder to respond to thermal stress. The results presented herein may contribute to the development of functional feed strategies that support the robustness, welfare, and sustainability of olive flounder aquaculture under ongoing climate-driven temperature variability.

## 2. Materials and Methods

### 2.1. Ethics Statement

The research was carried out in strict compliance with the ethical guidelines and regulatory standards established by the Animal Care and Use Committee of Pukyong National University, Republic of Korea (Approval No. PKNUIACUC-2022-48, approved on 19 September 2022). All procedures involving fish, including handling, rearing, sampling, and thermal stress challenges, were designed to minimize discomfort, stress, and mortality. Husbandry conditions were monitored throughout the experimental period to ensure compliance with national and institutional standards for the ethical use of aquatic animals in research. Every effort was made to reduce the number of animals used and to refine experimental protocols to safeguard the welfare of the juvenile olive flounder throughout the study.

### 2.2. Screening of Probiotics

The probiotic strains used in this study were isolated from the intestinal tract of healthy wild olive flounder collected from coastal waters in Korea and were therefore designated as host-associated strains. Following isolation, the bacterial isolates were putatively assigned to *Bacillus sonorensis*, *B. subtilis*, and *B. velezensis* based on 16S rRNA gene sequence analysis and phylogenetic clustering. Pure cultures of each strain were provided by the Molecular Biochemistry Laboratory, Department of Marine Biology, Pukyong National University (Busan, Republic of Korea). Preliminary in vitro assays were conducted with technical support from the Aquatic Animal Experimental Laboratory at Dong-eui University, Busan, Republic of Korea, to assess the growth responses of the isolated strains to fructo-oligosaccharides (FOS). De Man, Rogosa, and Sharpe (MRS) broth (Difco™ Lactobacilli MRS Broth, Becton, Dickinson and Company, Sparks, MD, USA; Cat. No. 288130), glucose-free MRS broth (MRS Broth w/o Glucose, Liofilchem S.r.l., Roseto degli Abruzzi, Italy; Cat. No. 610144), and MRS agar (Difco™ Lactobacilli MRS Agar, Becton, Dickinson and Company, Sparks, MD, USA; Cat. No. 288210) were prepared according to the manufacturers’ instructions and the formulation described by De Man et al. [40]. Fructo-oligosaccharide (FOS; ≥90% as FOS and inulin, from chicory; Sigma-Aldrich, Saint Louis, MO, USA; Cat. No. F8052) was dissolved in sterile distilled water to prepare the stock solution. MRS broth, glucose-free MRS broth, and MRS agar were sterilized by autoclaving according to the manufacturers’ instructions, whereas the FOS stock solution was filter-sterilized through a 0.22 µm sterile syringe filter (Millex™-GP, polyethersulfone membrane, 33 mm diameter; Merck Millipore, Darmstadt, Germany; Cat. No. SLGPR33RS) before supplementation.

Experimental media were prepared by supplementing the base broth with 5 g/L of fructo-oligosaccharide (FOS), whereas unsupplemented media served as controls to assess bacterial growth responses in the presence of FOS.

For agar-based assays, serially diluted bacterial cultures were spread onto MRS agar plates with or without FOS supplementation and incubated at 37 °C for 24 h, after which colony-forming units (CFUs) were subsequently enumerated to assess bacterial proliferation. For liquid culture assays, each probiotic strain at a concentration of 1 × 10^7^ CFU/mL was inoculated into glucose-free MRS broth supplemented with each prebiotic substrate, including glucose, galacto-oligosaccharide, FOS, inulin, chitosan, and β-glucan, and incubated at 37 °C in a shaking incubator (SI-600R, Jeio Tech Company Limited, Daejeon, Republic of Korea). Bacterial growth was monitored by measuring optical density at 600 nm (OD600) at 4 h intervals using a UV spectrophotometer (Shimadzu, Kyoto, Japan). Among the six prebiotics evaluated, FOS consistently supported the comparatively highest growth of all three host-associated *Bacillus* strains across the in vitro assays. In particular, cultures supplemented with FOS demonstrated higher colony-forming unit counts and optical density values over the incubation period compared with other prebiotic treatments, indicating greater bacterial growth responses. These preliminary in vitro findings supported the selection of FOS for inclusion in the experimental diets used in the feeding trial.

### 2.3. Experimental Diets Preparation

The composition and formulation of the experimental diets are presented in Table 1. Nine isonitrogenous (55% crude protein) and isolipidic (8% crude lipid) diets were formulated to meet the nutritional requirements of juvenile olive flounder. The control diet contained no probiotic or prebiotic additives. The experimental diets were categorized as follows: (1) single-strain combination diets containing one host-associated probiotic strain together with fructo-oligosaccharide (FOS), designated as *B. sonorensis* + FOS (AF), *B. subtilis* + FOS (BF), and *B. velezensis* + FOS (CF); (2) multi-strain combination diets containing combinations of two or three probiotic strains with FOS, designated as *B. sonorensis* + *B. subtilis* + FOS (ABF), *B. subtilis* + *B. velezensis* + FOS (BCF), *B. sonorensis* + *B. velezensis* + FOS (ACF), and *B. sonorensis* + *B. subtilis* + *B. velezensis* + FOS (ABCF); and (3) a prebiotic-only diet containing FOS (F). The final probiotic concentration in all probiotic-containing diets was adjusted to 1 × 10^7^ CFU g^−1^ diet, and FOS was included at 5 g kg^−1^ diet. Fish meal, squid liver meal, and soybean meal comprised the main protein sources, while fish oil served as the primary lipid source. Wheat flour and starch were included as carbohydrate sources.

All dry ingredients were weighed according to the formulation and mixed using a mechanical mixer (HYVM-1214, Hanyoung Food Machinery, Anyang, Republic of Korea). The water equivalent to 45% of the diet was gradually added and mixed for 15 min, followed by the addition of fish oil and probiotic culture broth, with an additional 15 min of mixing to ensure uniform distribution. The resulting mash was pelleted through a flat-die pelletizer equipped with a 2 mm die (SFD-GT, Shinsung, Anyang, Republic of Korea). Pellets were dried at 40 °C for 14 h in a grain drier (KE-010, Dongwon, Seoul, Republic of Korea) until the moisture content reached less than 10% and were subsequently stored at −20 °C in airtight containers until use.

### 2.4. Experimental Fish and Feeding Trial

Juvenile olive flounder used in this study were obtained from a certified commercial hatchery in Boryeong, Chungcheongnam-do, Rep. of Korea, and subsequently transported to the Feeds & Foods Nutrition Research Center (FFNRC) at Pukyong National University in Busan, Republic of Korea. Fish were acclimated to laboratory conditions for two weeks and were fed the control diet. Following acclimation, fish with an average initial body weight of 7.26 ± 0.04 g (mean ± SEM) were randomly assigned to 29 L acrylic tanks at a density of 18 fish per tank, with three replicate tanks allocated to each dietary treatment. The feeding trial lasted nine weeks. Water temperature was maintained within the optimal range for juvenile olive flounder using a temperature-regulating heat pump (DOV-887, Daeil, Busan, Republic of Korea), while constant aeration from an air blower maintained dissolved oxygen levels near saturation. Key water quality parameters, including pH, ammonia (NH_3_), nitrite (NO_2_^−^), and nitrate (NO_3_^−^), were monitored using a commercial test kit (API Fish Care, Chalfont, PA, USA). Additionally, dissolved oxygen and temperature were measured using a YSI DO meter (ProDPD/T, Yellow Springs, OH, USA). The water quality parameters maintained during the experiment are summarized as follows: NH_3_ 0.51 ± 0.03 mg/L, NO_2_^−^ 1.09 ± 0.08 mg/L, NO_3_^−^ 47.0 ± 3.5 mg/L, pH 7.49 ± 0.02, DO 7.32 ± 0.02 mg/L, and temperature 19.7 ± 0.1 °C. All tanks were supplied with filtered seawater at a rate of 2.7 L min^−1^, and mechanical filtration using filter wool and a protein skimmer was applied to remove suspended particulates and reduce dissolved nitrogenous waste. Experimental diets were administered manually twice daily (09:00 and 17:00) at approximately 3% of body weight, with rations adjusted according to biweekly assessments of growth and mortality. Uneaten feed and fecal matter were removed twice daily by siphoning, and additional daily water exchange was performed to maintain optimal rearing conditions.

### 2.5. Lethal Temperature Challenge

Upon completion of the nine-week feeding trial, a subsample of seven to ten fish from each tank was randomly selected to evaluate survival under a lethal high-temperature challenge. The number of fish used for the lethal challenge varied slightly among tanks because of differences in the number of fish remaining after the feeding trial and allocation of fish for other terminal analyses. All available fish assigned to the lethal challenge were included. Although this resulted in unequal fish numbers among tanks, the number of replicate tanks remained balanced across treatments, with three tanks per dietary treatment. Time-dependent survival data were analyzed using Kaplan–Meier survival analysis with the log-rank test, which can accommodate unequal numbers of individuals among groups; therefore, this variation was not expected to introduce systematic bias, although it may have slightly reduced the precision of survival estimates. To reduce the possibility of tank-related confounding effects, the challenge was conducted in the same 29 L acrylic tanks used during the rearing phase. Water temperature was increased from the rearing temperature to 30.5 °C at a controlled rate of 0.5 °C every 30 min using a heat pump. The target temperature of 30.5 °C was selected to impose a lethal thermal challenge above the optimal rearing range of juvenile olive flounder while allowing time-dependent mortality to be recorded rather than causing immediate mass mortality. A gradual increase of 0.5 °C every 30 min was used to standardize thermal exposure across tanks and reduce abrupt handling- or shock-related effects during the lethal challenge. Once the target temperature was reached, fish were monitored visually at 6 h intervals. Individuals exhibiting complete cessation of opercular movement were considered dead, immediately removed from the tank, and measured for body weight and total length, and mortality was recorded at each observation point. Water temperature was continuously monitored throughout the challenge using a HOBO Water Temperature Pro v2 data logger (U22-001, Onset, Bourne, MA, USA) to ensure accurate thermal profiling and consistency among tanks. This experimental design allowed assessment of cumulative mortality and survival responses among dietary treatments under standardized thermal-challenge conditions.

### 2.6. Acute Temperature Stress Exposure

Following the nine-week feeding trial, three olive flounders from each tank were randomly selected for the acute thermal challenge and transferred into perforated plastic cages. The cages were then placed in a 216 L tank used for the acute high-temperature exposure. Fish were abruptly exposed to 30 °C for 2 h using a 2 kW titanium submersible heater (Unitech, Incheon, Republic of Korea). The acute exposure temperature of 30 °C and the 2 h exposure duration were selected to induce short-term physiological and molecular heat-stress responses without causing immediate mortality. The subsequent 2 h recovery period at 19.7 °C was selected to capture early post-stress physiological and transcriptional responses after return to the rearing temperature, while minimizing the possibility that delayed compensatory responses or secondary recovery processes would obscure the immediate effects of acute heat exposure. Because stress-related plasma and gene-expression responses may vary with recovery duration, the results of this study should be interpreted as early recovery-phase responses rather than as a complete temporal profile of post-stress regulation. Prior to the experiment, heater settings were calibrated in a preliminary trial to ensure that the target temperature could be reliably achieved and maintained. Water temperature during the exposure period was continuously monitored using the temperature data recorder (U22-001, Onset, Bourne, MA, USA) to ensure thermal stability and consistency across trials. After the 2 h heat exposure, fish were rapidly transferred to a recovery tank maintained at 19.7 °C and allowed to recover under controlled conditions for an additional 2 h prior to sampling. This protocol allowed for a standardized assessment of acute physiological and molecular responses to thermal stress among dietary treatments.

### 2.7. Growth Performance

At the end of the nine-week feeding trial, the fish were fasted for a 24 h period to ensure gut clearance prior to morphometric measurements. The total number of fish and the total biomass in each tank were recorded to assess growth performance parameters, including final body weight (FBW), weight gain (WG), specific growth rate (SGR), feed efficiency (FE), and survival rate (SR). Subsequently, three fish from each tank were randomly selected for comprehensive morphometric and tissue analyses. Fish were anesthetized with 2-phenoxyethanol (200 ppm for 5 min), and their total length and body weight were measured. Following anesthesia, the fish were dissected, and major tissues, including the brain, gills, kidney, and liver, were collected for subsequent molecular analyses. The somatic indices were calculated to evaluate the physiological condition of the fish following the feeding trial. The indices comprised the hepatosomatic index (his), viscerosomatic index (VSI), and condition factor (CF), which are widely used metrics for assessing organ size, energy allocation, and overall body condition in teleosts. Each parameter was derived using standard morphometric relationships according to the following equations

WG (%) = [final weight (g) − initial weight (g)]/initial weight (g) × 100.

SGR (%/day) = [ln (final weight (g)) − ln (initial weight (g))]/days × 100.

FE (%) = (final weight (g) − initial weight (g))/feed consumed (g) × 100.

SR (%) = (final number of fish/initial number of fish) × 100.

HSI (%) = liver weight (g)/body weight (g) × 100.

VSI (%) = viscera weight (g)/body weight (g) × 100.

CF = wet weight (g)/total length (cm)^3^ × 100

### 2.8. Diet and Fish Whole Body Proximate Composition Analysis

The proximate composition analyses were performed on all nine experimental diets and on whole-body samples collected from three randomly selected fish per tank at the end of the feeding trial. The analytical procedures were conducted in accordance with the standardized methodologies established by the Association of Official Analytical Chemists [41]. Prior to analysis, samples were freeze-dried for 72 h using a laboratory freeze dryer (Advantage 2.0, VirTis, New York, NY, USA) and subsequently homogenized with a mortar and pestle to obtain a homogeneous powder. The crude ash content was determined using the direct ashing method, wherein approximately 1 g of finely ground sample was combusted in a muffle furnace at 550 °C for 3 h. The moisture content was assessed using the atmospheric oven-drying method by heating approximately 1 g of sample at 135 °C for 3 h. Crude protein was measured using the Kjeldahl technique (nitrogen × 6.25) with an automated digestion and distillation apparatus (Buchi B324/435/412, Flawil, Switzerland). The crude lipid content was determined using the Soxhlet extraction method (Soxtec System 1046, Tecator AB, Höganäs, Sweden) in accordance with the chloroform–methanol extraction method described by Folch et al. [42]. These analyses provided a standardized evaluation of macronutrient composition to assess the nutritional quality of the experimental diets and the whole-body composition of fish across different treatments.

### 2.9. Plasma Metabolites Analysis

At the end of the feeding trial, three olive flounder were randomly selected from each tank for plasma biochemical analysis. Plasma samples from individual fish were analyzed separately, and the mean value of the three fish from each tank was used as the experimental unit for statistical analysis. Thus, each dietary treatment had three replicate tank-level values (*n* = 3). Fish were anesthetized, and blood samples were obtained from the caudal vein using 1 mL syringes pre-treated with dipotassium ethylenediaminetetraacetic acid (K_2_-EDTA) to inhibit coagulation. Approximately 1 mL of whole blood was collected from each fish and transferred into 3 mL siliconized vacuum tubes containing 5.4 mg of K_2_-EDTA (Becton Dickinson and Company, Franklin Lakes, NJ, USA) to ensure consistent anticoagulant conditions across all samples. Blood samples were then aliquoted into 1.5 mL microcentrifuge tubes and centrifuged at 11,000× *g* for 5 min at 4 °C using a refrigerated microcentrifuge (Micro 17TR, Hanil Science Industrial Company Limited, Incheon, Republic of Korea) to isolate plasma. The plasma samples were stored at −84 °C in an ultra-low temperature freezer (MDF-U74V, PHC Holdings Corporation, Tokyo, Japan) until biochemical analysis. Plasma metabolites, such as glucose (GLU), total cholesterol (TCHO), triglycerides (TG), total protein (TP), glutamic oxaloacetic transaminase (GOT), and glutamic pyruvic transaminase (GPT), were measured using a dry chemistry analyzer (Fuji DRI-CHEM 4000i, FUJIFILM Corporation, Tokyo, Japan) with corresponding FujiFilm DRI-CHEM SLIDE reagent kits. These biochemical indicators were used to assess metabolic status, liver function, and overall physiological condition across dietary treatments.

### 2.10. Antioxidant Enzyme, Immune Reaction, and Stress-Related Parameters

Plasma samples collected from the experimental fish were analyzed to assess antioxidant capacity, innate immune function, and stress-related responses. Concentrations of glutathione peroxidase (GPx), superoxide dismutase (SOD), immunoglobulin M (IgM), lysozyme (LZM), cortisol, and heat shock protein 70 (HSP70) were measured with commercially available enzyme-linked immunosorbent assay (ELISA) kits (CUSABIO, Houston, TX, USA). All analyses were conducted manually in accordance with the manufacturer’s specified protocols, encompassing standard preparation, reagent addition, incubation steps, and washing cycles. Following completion of the ELISA reactions, absorbance was recorded at 450 nm within 5 min using a microplate reader (AMR-100, Allsheng, Hangzhou, China) to maintain consistency and avoid signal degradation. Each biomarker was measured in technical duplicate for each plasma sample. The duplicate readings were averaged to obtain one value for each individual fish. Individual fish values within each tank were then averaged to generate one representative tank-level value for statistical analysis. Thus, the tank, rather than the individual fish or technical duplicate, was used as the experimental unit. These measurements were used as indicators of oxidative status, innate immune function, and stress-related responses across dietary treatments.

### 2.11. Histological Analysis

For histological assessment, midgut segments of approximately 1 cm were collected from three fish per tank and promptly fixed in 10% neutral-buffered formalin for 14 days prior to being transferred to 70% ethanol for preservation. The 14-day fixation period was used to ensure complete fixation of all intestinal tissues before batch processing, and the same fixation duration was applied consistently to all samples across dietary treatments. Although prolonged formalin fixation may influence tissue morphology or staining intensity, all samples were processed under identical fixation, dehydration, embedding, sectioning, and staining conditions to minimize treatment-related processing bias. All histology procedures were performed with technical assistance from the Physiology Laboratory at Pukyong National University. Fixed tissues underwent dehydration using a sequential ethanol series (70%, 80%, 90%, 95%, and 100%), with each stage lasting 1 h to ensure the complete removal of water. Following standard paraffin-embedding procedures, dehydrated samples were subsequently cleared in xylene and infiltrated with molten paraffin. A rotary microtome (CUT 4055, MicroTec, Wetzlar, Germany) was used to section paraffin blocks at a thickness of 4–5 µm. The sections were then mounted onto glass slides and dried on a heating plate at 50 °C before staining. Histological sections were stained with Harris hematoxylin and eosin (H&E) and mounted using Canada balsam. Stained slides were examined using a light microscope (OLYMPUS BX41, Tokyo, Japan) equipped with a digital imaging system (DIXI Optics, Daejeon, Republic of Korea). The images were recorded and analyzed with Motic Image Plus 2.0 software (Motic Instruments Inc., Fujian, China). Morphometric parameters were measured to assess intestinal morphology. Villus length (VL; µm) was quantified as the distance from the highest point of the villus to its base, while muscular thickness (MT; µm) was measured as the distance from the circular muscle layer to the intestinal epithelium. For each specimen, ten measurements were recorded per cross-section and averaged to derive representative values for each fish.

### 2.12. Gene Expression

Following the acute temperature exposure and 2 h recovery period described in Section 2.6, three fish from each tank were used for qPCR analysis, with three replicate tanks per dietary treatment, resulting in nine fish per treatment for each tissue. Brain, gill, liver, and kidney tissues were collected from each fish, snap-frozen in liquid nitrogen, and immediately stored at −84 °C until analysis. To minimize RNA degradation, all tissue samples were handled under RNase-free conditions during sampling, homogenization, and extraction procedures. Tissue samples (≤100 mg) from the brain, gills, liver, and kidney were homogenized in RiboEx™ using a bead mill homogenizer (Bioprep-6, Hangzhou Allsheng Instruments Company Limited, Hangzhou, China). Total RNA was then extracted using the Hybrid-R™ total RNA extraction kit (GeneAll Biotechnology Company Limited, Hanam, Republic of Korea; Cat. No. 305-101) according to the manufacturer’s protocol.

A NanoDrop Lite Plus spectrophotometer (Thermo Fisher Scientific, Waltham, MA, USA) was used to determine RNA concentration and purity by measuring absorbance at 260 and 280 nm. The A260/A280 ratios for the RNA extracted from the brain, gills, liver, and kidney were 2.15 ± 0, 2.18 ± 0, 2.15 ± 0.01, and 2.15 ± 0, respectively, indicating acceptable RNA purity. Only RNA samples showing acceptable purity were used for downstream cDNA synthesis and qPCR analysis. Complementary DNA (cDNA) was synthesized using the PrimeScript™ 1st Strand cDNA Synthesis Kit (Takara Bio Incorporation, Kusatsu, Japan; Cat. No. 6110A) according to the manufacturer’s instructions. The synthesized cDNA was stored at −20 °C until real-time PCR analysis. The resulting cDNA was used as the template for quantitative real-time PCR (qPCR) to quantify transcript abundance in each tissue. The mRNA expression levels of heat shock protein 60 (*HSP60*), heat shock protein 70 (*HSP70*), heat shock protein 90α (*HSP90α*), heat shock protein 90β (*HSP90β*), glucose-6-phosphatase (*G6pase*), and AMP-activated protein kinase beta (*AMPKβ*) were analyzed. β-actin was used as the reference gene based on its previous application in qPCR studies of olive flounder, and the successful amplification specificity was confirmed by melting curve analysis in the present study. However, because β-actin stability was not independently evaluated against additional candidate reference genes under the present acute heat-stress conditions, this should be considered when interpreting the relative gene-expression data.

Quantitative real-time PCR was performed using TB Green^®^ Premix Ex Taq™ II (Tli RNaseH Plus; Takara Bio Inc., Kusatsu, Japan; Cat. No. RR820A) on a StepOne Real-Time PCR System (Applied Biosystems, Thermo Fisher Scientific, Waltham, MA, USA). The primer sequences used in this study are shown in Table 2. Each reaction was prepared using the SYBR Green-based premix, gene-specific forward and reverse primers, cDNA template, and nuclease-free water. All reactions were run under identical amplification conditions. The qPCR cycling conditions were as follows: initial denaturation at 95 °C for 30 s, followed by 40 cycles of denaturation at 95 °C for 4 s and annealing/extension at 60 °C for 30 s. At the end of the reaction, a melting curve analysis was performed to ensure the specificity of the amplified products. A single distinct melting peak was considered indicative of specific amplification.

All qPCR reactions for each target gene were performed in triplicate. These triplicates represented technical replicates for each biological sample. The relative mRNA expression levels were normalized to *β-actin* and calculated using the 2^−ΔΔCt^ method. For each sample, the Ct values of the target genes were first normalized to the Ct value of *β-actin* to obtain the ΔCt value and then compared with the corresponding control group to calculate ΔΔCt. Relative expression levels were expressed as fold changes relative to the control. The data were analyzed with StepOne Software version 2.0 (Applied Biosystems, Waltham, MA, USA).

### 2.13. Statistical Analysis

All experimental tanks were assigned to dietary treatments based on a completely randomized design. The tank was considered the experimental unit for all statistical analyses. For parameters measured from multiple fish within each tank, individual fish values were averaged to obtain one representative tank-level value before statistical analysis. For assays performed in technical duplicate or triplicate, technical replicate values were first averaged to obtain one biological sample value before calculating tank-level means. Data were assessed for normality and homogeneity of variance using the Shapiro–Wilk test and Levene’s test, respectively. Growth performance, somatic indices, whole-body proximate composition, plasma biochemical parameters, antioxidant and immune-related indicators, intestinal morphology, and relative gene expression data were analyzed by one-way analysis of variance (ANOVA) to determine the effects of dietary treatment. For qPCR data, statistical analyses were performed using ΔCt values, while relative expression levels were presented as fold changes calculated using the 2^−ΔΔCt^ method. When significant differences were detected (*p* < 0.05), Tukey’s honestly significant difference (HSD) test was used for multiple comparisons among treatment means. Time-dependent survival data from the lethal temperature challenge were analyzed using the Kaplan–Meier survival analysis, and differences in survival curves among dietary treatments were evaluated using the log-rank test. One-way ANOVA was applied only to endpoint variables and was not used for the time-dependent survival data. All statistical analyses were performed using SAS software version 9.4 (SAS Institute Inc., Cary, NC, USA). Results are presented as mean ± standard error of the mean (SEM), and statistical significance was set at *p* < 0.05.

## 3. Results

### 3.1. Growth Performance

The effects of dietary supplementation with single- and multi-strain host-associated *Bacillus*–FOS formulations on the growth performance and morphological indices of juvenile olive flounder during the nine-week feeding trial are presented in Table 3. No statistically significant differences were observed among dietary treatments for IBW, FBW, WG, FE, SGR, SR, CF, HSI, or VSI (*p* > 0.05). Across dietary treatments, final body weight ranged from 39.6 to 42.5 g, weight gain from 444% to 506%, feed efficiency from 122% to 135%, specific growth rate from 2.73% to 2.91% day^−1^, and survival from 90.7% to 100%. Similarly, condition factor, hepatosomatic index, and viscerosomatic index ranged from 1.11 to 1.26, 1.36% to 2.11%, and 2.86% to 3.88%, respectively. These results indicate that the observed numerical variation among treatments did not result in statistically significant treatment-related differences in growth performance or morphological indices under the present rearing conditions.

### 3.2. Whole Body Proximate Composition

The whole-body proximate composition of juvenile olive flounder following the nine-week feeding trial is summarized in Table 4. No statistically significant treatment effects were observed for moisture, crude protein, crude lipid, or crude ash content (*p* > 0.05). Moisture content ranged from 72.4% to 74.6%, crude protein from 18.9% to 20.7%, crude lipid from 2.71% to 3.00%, and crude ash from 3.59% to 4.12% across dietary treatments. Although minor numerical variation was observed, the data did not provide statistical evidence that dietary supplementation with host-derived *Bacillus* strains and/or FOS altered whole-body macronutrient composition during the nine-week feeding period.

### 3.3. Plasma Metabolites

Plasma biochemical parameters measured at the end of the nine-week feeding trial are shown in Table 5. Dietary treatment did not significantly affect GOT, GPT, GLU, TCHO, TP, or TG concentrations (*p* > 0.05). Across treatments, GOT, GPT, GLU, TCHO, TP, and TG ranged from 17.0 to 22.0 U/L, 17.3–21.7 U/L, 19.7–39.0 mg/dL, 116–140 mg/dL, 2.97–3.23 g/dL, and 180–272 mg/dL, respectively. These data indicate that the dietary treatments did not produce detectable changes in circulating biochemical indicators under normal rearing conditions.

### 3.4. Antioxidant Enzyme, Immune Reaction, and Stress-Related Parameters

The plasma antioxidant, immune-related, and stress-related parameters measured after the nine-week feeding trial are presented in Table 6. No statistically significant differences were demonstrated among dietary treatments for GPx, SOD, IgM, LZM, HSP70, or cortisol concentrations (*p* > 0.05). Across dietary treatments, GPx ranged from 197 to 370 mU/mL, SOD from 512 to 640 ng/mL, IgM from 1.42 to 1.57 µg/mL, LZM from 4.72 to 9.73 µg/mL, HSP70 from 19.8 to 33.5 pg/mL, and cortisol from 2.70 to 4.03 ng/mL. These numerical differences did not correspond to statistically significant treatment-related variation. Therefore, under the present experimental conditions, dietary supplementation with host-associated *Bacillus* strains and/or FOS was not associated with detectable changes in the measured antioxidant, immune-related, or stress-related plasma indicators.

### 3.5. Intestinal Histology

**Figure 1:**
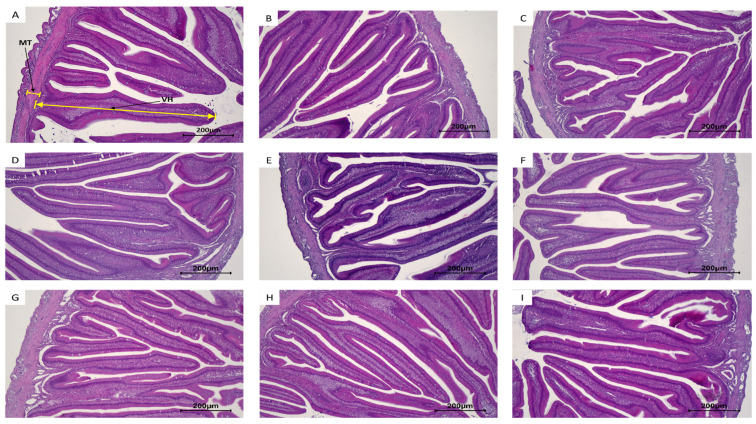
Representative histological sections of the midgut of juvenile olive flounder (*Paralichthys olivaceus*) fed nine experimental diets for nine weeks. Sections were stained with hematoxylin and eosin (H&E) and observed at 100× magnification. VH and MT represent villus height and muscular thickness, respectively. (**A**) CON, control diet; (**B**) AF, *Bacillus sonorensis* + fructo-oligosaccharide (FOS); (**C**) BF, *B. subtilis* + FOS; (**D**) CF, *B. velezensis* + FOS; (**E**) ABF, *B. sonorensis* + *B. subtilis* + FOS; (**F**) BCF, *B. subtilis* + *B. velezensis* + FOS; (**G**) ACF, *B. sonorensis* + *B. velezensis* + FOS; (**H**) ABCF, *B. sonorensis* + *B. subtilis* + *B. velezensis* + FOS; (**I**) F, FOS-only diet. The black arrows indicate the measured parameters (mucosal thickness: MT; villus height: VH), and the yellow arrows indicate the corresponding measurement directions and distances.

Histological observations and morphometric measurements of the midgut of juvenile olive flounder after the nine-week feeding trial are summarized in Table 7 and Figure 1. No statistically significant differences were revealed among dietary treatments for villus height or muscular thickness (*p* > 0.05). Villus height ranged from 734 to 906 µm, while muscular thickness ranged from 48.1 to 63.6 µm across dietary treatments. Although numerical variation was observed, the intestinal morphometric parameters were statistically comparable among fish fed the control diet, single-strain synbiotic diets, multi-strain synbiotic diets, and the FOS-only diet. Representative H&E-stained sections showed no evident treatment-related alterations in intestinal tissue organization or epithelial structure. These results indicate that the tested dietary treatments did not produce detectable differences in midgut morphology under the present rearing conditions.

### 3.6. Lethal Temperature Challenge

**Figure 2:**
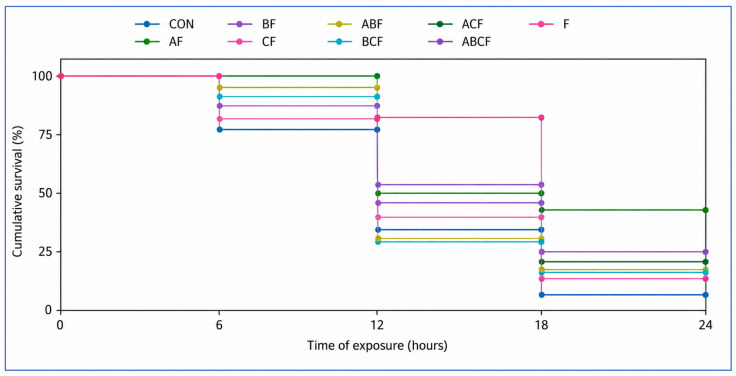
Cumulative survival (%) of juvenile olive flounder subjected to a lethal high-temperature challenge. Fish were exposed to increasing water temperature up to 30.5 °C, and cumulative survival was monitored over time. Values are presented as mean ± SEM from three replicate tanks per treatment. Differences among dietary treatments were analyzed using Kaplan–Meier survival analysis followed by the log-rank test. CON, control; AF, *Bacillus sonorensis* + fructo-oligosaccharide (FOS); BF, *B. subtilis* + FOS; CF, *B. velezensis* + FOS; ABF, *B. sonorensis* + *B. subtilis* + FOS; BCF, *B. subtilis* + *B. velezensis* + FOS; ACF, *B. sonorensis* + *B. velezensis* + FOS; ABCF, *B. sonorensis* + *B. subtilis* + *B. velezensis* + FOS; F, FOS-only diet.

The cumulative survival of juvenile olive flounder subjected to the lethal-temperature challenge is illustrated in Figure 2. During the early phase of exposure, survival remained relatively high across all dietary treatments; however, mortality progressively increased as the thermal challenge continued. No statistically significant differences in cumulative survival were identified among dietary treatments during the lethal temperature challenge (*p* > 0.05). At 24 h post-exposure, fish fed the multi-strain diets ACF and ABCF showed numerically higher survival, approximately 16.2% and 16.7%, respectively, whereas the remaining treatments showed lower numerical survival values. However, these differences were not statistically significant, and the survival curves should therefore be interpreted as a numerical tendency rather than evidence of a definitive dietary effect. Overall, the lethal temperature challenge revealed no significant treatment-related differences in cumulative survival under the conditions tested.

### 3.7. Acute Temperature Exposure

Plasma biochemical responses after acute temperature exposure are summarized in Table 8. Dietary treatment did not significantly affect hematocrit, GOT, GPT, GLU, TCHO, TP, TG, or cortisol after acute heat exposure (*p* > 0.05). Across treatments, hematocrit, GOT, GPT, GLU, TCHO, TP, TG, and cortisol ranged from 17.8 to 19.2%, 21.7–50.7 U/L, 18.7–27.3 U/L, 24.0–55.3 mg/dL, 84.0–111 mg/dL, 2.50–3.00 g/dL, 119–161 mg/dL, and 7.00–11.3 ng/dL, respectively. These results do not provide evidence that the dietary treatments altered plasma biochemical responses following the acute heat-stress protocol.

**Figure 3:**
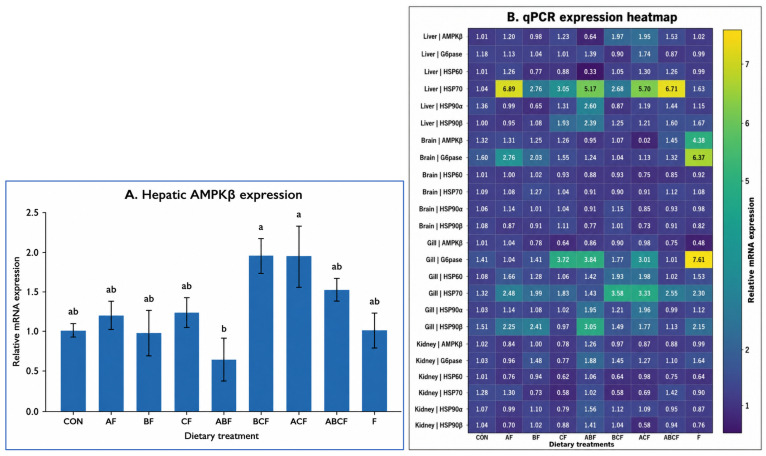
Relative mRNA expression of heat-shock- and energy-metabolism-related genes in juvenile olive flounder (*Paralichthys olivaceus*) after acute heat exposure. (**A**) Hepatic *AMPKβ* expression, the only transcript showing a significant dietary treatment effect. Different letters indicate significant differences among dietary treatments based on one-way ANOVA followed by Tukey’s HSD test (*p* < 0.05). (**B**) Heatmap showing the relative expression patterns of *AMPKβ*, *G6pase*, *HSP60*, *HSP70*, *HSP90α*, and *HSP90β* in liver, brain, gill, and kidney. Values are expressed as fold changes relative to the control group using the 2^−ΔΔCt^ method. Statistical analyses were performed using ΔCt values. CON, control diet; AF, *Bacillus sonorensis* + fructo-oligosaccharide (FOS); BF, *B. subtilis* + FOS; CF, *B. velezensis* + FOS; ABF, *B. sonorensis* + *B. subtilis* + FOS; BCF, *B. subtilis* + *B. velezensis* + FOS; ACF, *B. sonorensis* + *B. velezensis* + FOS; ABCF, *B. sonorensis* + *B. subtilis* + *B. velezensis* + FOS; F, FOS-only diet.

Among the genes analyzed following acute temperature exposure, hepatic *AMPKβ* was the only transcript showing a significant dietary treatment effect (*p* = 0.0307; Figure 3A). Hepatic *AMPKβ* expression was significantly higher in the BCF and ACF groups than in the ABF group, whereas the remaining dietary treatments showed intermediate values and did not differ significantly from either group. In contrast, no significant dietary treatment effects were detected for hepatic *G6pase*, *HSP60*, *HSP70*, *HSP90α*, or *HSP90β* expression. In the brain, gill, and kidney, none of the analyzed heat shock- or energy metabolism-related genes differed significantly among dietary treatments (Figure 3B).

Overall, these results indicate that the transcriptional response to the dietary treatments after acute heat exposure was limited. Although hepatic *AMPKβ* showed a significant dietary treatment effect, this response was not accompanied by significant changes in hepatic G6pase or heat shock protein genes, nor by significant transcriptional differences in the brain, gill, or kidney. Therefore, hepatic *AMPKβ* represented the only significant molecular response detected under the present experimental conditions. The complete numerical qPCR data, including mean ± SEM values and statistical annotations for each tissue and target gene, are provided in Table 9.

## 4. Discussion

The principal finding of this study was that dietary host-derived *Bacillus*–FOS formulations were well tolerated by juvenile olive flounder but did not produce broad improvements in growth performance, basal physiological status, intestinal morphology, or thermal stress resilience under the present experimental conditions. Although hepatic *AMPKβ* was significantly affected by dietary treatment after acute heat exposure, this response was not accompanied by consistent changes in plasma metabolites, heat shock protein expression, or survival. Therefore, the overall interpretation of the present dataset is that the tested formulations produced limited and endpoint-specific responses rather than a generalized improvement in thermal stress tolerance. In the present study, all experimental diets were formulated with a high inclusion level of fishmeal (60%), consistent with protein-rich formulations commonly used in commercial olive flounder aquafeeds [50,51]. This formulation was intended to provide a highly digestible diet with an adequate amino acid profile, thereby supporting stable baseline growth, as high-fishmeal diets are commonly used in olive flounder aquafeeds [50,51]. Under these nutritionally favorable conditions, dietary supplementation with host-derived *Bacillus* strains and FOS, either as single- or multi-strain formulations, did not significantly improve growth performance, feed efficiency, survival, or morphometric indices in juvenile olive flounder. Although dietary *Bacillus* spp. have been reported to improve growth, feed utilization, digestive function, and health status in several aquaculture species [52,53,54,55,56,57,58,59,60,61,62], no growth-promoting effect was detected under the present dietary conditions. Because digestive enzyme activity, gut microbial composition, nutrient digestibility, and probiotic colonization were not directly evaluated in the present study, the absence of growth improvement was interpreted primarily from the measured endpoints, including growth performance, whole-body composition, plasma biochemical indicators, and intestinal morphology. The lack of response may be partly explained by the high nutritional quality of the basal diet, which likely reduced the physiological scope for additional improvement. In a diet already containing 60% fishmeal, growth performance, nutrient supply, and baseline physiological status may have been sufficiently supported in the control group, making additional probiotic- or synbiotic-mediated benefits difficult to detect. Therefore, the limited response observed in the present study should be interpreted in relation to the high-fishmeal dietary background, and future studies should evaluate these formulations under more nutritionally challenging conditions, such as lower fishmeal inclusion, higher plant-protein replacement, or reduced dietary digestibility.

The dietary treatments also did not significantly alter whole-body composition, plasma biochemical parameters, antioxidant and immune-related indicators, cortisol, or intestinal morphology under normal rearing conditions. Plasma metabolites and enzyme activities are widely used as indicators of metabolic stability, hepatic function, and stress-induced homeostatic disruption in fish [63,64,65,66,67]. In particular, GOT and GPT are commonly regarded as non-specific indicators of hepatic or cellular membrane disturbance because their release into circulation can be associated with cellular damage, oxidative imbalance, or inflammatory responses [68,69,70,71,72]. In the present study, the comparable GOT, GPT, GLU, TCHO, TP, and TG values among dietary groups indicate that the host-associated *Bacillus*-FOS formulations did not produce detectable changes in metabolic or hepatic status during the feeding trial. Similarly, the absence of significant differences in GPx, SOD, IgM, LZM, HSP70, and cortisol suggests that antioxidant capacity, innate immune status, and basal stress condition were not markedly affected by the dietary treatments. Importantly, these results also indicate that the tested formulations did not adversely affect physiological homeostasis or intestinal morphology under normal rearing conditions. Thus, although the dietary treatments did not enhance baseline physiological indicators, they appeared to be well tolerated by juvenile olive flounder during the nine-week feeding period.

Following acute temperature exposure, olive flounder exhibited clear physiological responses characterized by increased GLU, GOT, GPT, and cortisol, together with reduced TP, TCHO, and TG. These responses indicate that acute thermal stress induced rapid metabolic and neuroendocrine adjustments. Increased GOT and GPT activities during stressful events may reflect elevated metabolic demands and altered hepatic or cellular membrane status [73]. Similar increases in plasma GOT, GPT, and GLU have been reported in barbel carp (*Luciobarbus capito*) exposed to elevated temperatures [74]. Blood GLU is a key energy substrate, particularly under conditions of heightened energetic demand [75], and stress-induced hyperglycemia is a well-known secondary stress response in teleosts, largely mediated by cortisol-dependent gluconeogenesis and hepatic energy mobilization [76,77]. Cortisol, the principal corticosteroid hormone involved in the teleost stress response [78,79], is regulated mainly through the hypothalamus–pituitary–interrenal axis during environmental stress [80]. Its elevation under acute thermal stress promotes gluconeogenesis, glycogenolysis, and mobilization of stored energy reserves [81,82,83], making it a widely used biomarker for environmental and husbandry-related stress in fish [84,85]. The increased cortisol response observed in this study is consistent with previous findings in marine fish exposed to temperature stress, including gilthead seabream (*Sparus aurata*) [86]. However, despite these clear stress-related physiological shifts, dietary treatment did not significantly modify plasma biochemical or cortisol responses after acute heat exposure. This suggests that the tested host-associated *Bacillus*-FOS formulations had limited capacity to alter short-term systemic stress responses under the present acute challenge conditions. The abrupt thermal exposure used in this study was designed as a standardized acute heat-stress model rather than as a complete simulation of all temperature fluctuations occurring under commercial aquaculture conditions. In field conditions, water temperature often increases more gradually over diel or seasonal time scales, although rapid temperature changes may occur during heatwave events, shallow-water or tank-based culture, water-exchange events, or other operational disturbances. Therefore, the 30 °C exposure for 2 h is useful for comparing short-term physiological and molecular responses among dietary treatments, but it may not fully represent chronic or fluctuating thermal stress experienced in commercial olive flounder farms. Future studies should include gradual warming, diel temperature fluctuation, and prolonged heat-stress models to better evaluate field-relevant thermal resilience. Because only one recovery time point was evaluated, the present study cannot determine whether dietary effects would emerge at earlier or later recovery phases. Future studies using multiple post-stress sampling points would help clarify the temporal dynamics of physiological and molecular recovery after acute heat exposure.

At the molecular level, most heat-shock- and energy-metabolism-related genes examined after acute thermal exposure were not significantly affected by dietary treatment. Heat shock proteins are central components of the cellular stress response and contribute to protein stabilization, refolding of denatured proteins, and protection against oxidative and thermal damage [87,88,89]. Their expression is frequently elevated in fish exposed to increased temperatures [90,91]. In the present study, however, *HSP60*, *HSP70*, *HSP90α*, and *HSP90β* did not differ significantly among dietary treatments, suggesting that the dietary formulations did not substantially modify *HSP*-related transcriptional responses following acute heat exposure. Similarly, G6pase, a key enzyme involved in glycogenolysis and gluconeogenesis during periods of elevated energy demand [92,93], was not significantly affected by dietary treatment. Although the observed increase in plasma GLU after acute temperature exposure suggests activation of systemic energy mobilization, the absence of dietary effects on *G6pase* expression indicates that the treatments did not produce detectable differences in this hepatic glucose-regulatory pathway under the experimental conditions tested.

In contrast to the relatively stable expression patterns of *HSPs* and *G6pase*, hepatic *AMPKβ* was the only molecular marker showing a significant dietary treatment effect after acute temperature exposure. AMPK is a highly conserved regulator of cellular and organismal energy homeostasis and is activated under metabolic stress to restore ATP balance through coordinated regulation of lipid, carbohydrate, and protein metabolism [94,95]. In the present study, *AMPKβ* expression was higher in the BCF and ACF groups than in the ABF group, while the remaining treatments showed intermediate values. This result may suggest that certain multi-strain *Bacillus*–FOS formulations were associated with differential regulation of hepatic energy-sensing pathways. However, this finding should be interpreted cautiously because it was not accompanied by consistent treatment-related differences in plasma metabolites, *HSP* expression, *G6pase* expression, or other physiological endpoints [96]. Therefore, although hepatic *AMPKβ* represents the primary molecular response detected in this study, its biological relevance remains uncertain and requires further investigation under experimental conditions designed to clarify energy metabolism and microbial function more directly. Several plasma biochemical and gene-expression variables exhibited relatively high inter-tank variability. Given that the experimental design included three replicate tanks per dietary treatment, the statistical power to detect modest dietary effects may have been limited. Accordingly, treatment-related numerical variation in plasma parameters, gene expression, and survival was not considered sufficient evidence of biological effects unless supported by statistical significance. In this context, the significant hepatic *AMPKβ* response represents an isolated molecular finding, whereas the non-significant survival tendency was not consistently supported by corresponding changes in other physiological or molecular endpoints.

The lethal temperature challenge showed numerically higher survival in fish fed some multi-strain synbiotic diets, particularly ACF and ABCF, but these differences were not statistically significant. Therefore, this pattern should be regarded as a preliminary tendency rather than evidence of improved thermal tolerance. The lack of significant survival differences may be related to the intensity of the challenge, the relatively small number of replicate tanks, or the possibility that the dietary treatments produced only modest physiological effects under the conditions tested. Collectively, the predominance of non-significant responses across growth, plasma biochemistry, antioxidant and immune indicators, intestinal morphology, survival, and most gene-expression endpoints suggests that the functional effect of the tested *Bacillus*–FOS formulations was limited under the present high-fishmeal dietary background and acute thermal challenge model. The numerical survival tendency observed in ACF and ABCF, together with the isolated hepatic *AMPKβ* response, may indicate a potential direction for further investigation; however, these findings were not sufficiently supported by the broader physiological and molecular dataset to confirm enhanced thermal resilience. Because gut microbial composition, microbial metabolites, and probiotic colonization were not assessed in the present study, the microbial basis of the observed *AMPKβ* response and numerical survival tendency cannot be determined from the current dataset. Accordingly, interpretations involving gut microbiota-mediated effects, short-chain fatty acid production, or related metabolic signaling pathways should be considered speculative. Future studies incorporating microbiome profiling, targeted metabolomics, short-chain fatty acid analysis, and strain colonization assessment are needed to clarify whether host-associated *Bacillus*–FOS formulations can modulate thermal-stress resilience in olive flounder. In addition, because the probiotic isolates were assigned to species level based on 16S rRNA gene sequence analysis, future studies should confirm their taxonomic identity using additional housekeeping genes, multilocus sequence analysis, or whole-genome sequencing to improve species-level resolution within the genus *Bacillus*. Furthermore, evaluating these formulations under low-fishmeal or plant-based diets, chronic or fluctuating heat-stress regimes, and combined environmental stressors may help determine whether dietary context and stress duration influence their functional efficacy.

## 5. Conclusions

The present study showed that dietary host-associated *Bacillus*–FOS formulations did not significantly improve growth performance, basal physiological indicators, intestinal morphology, or most heat-shock- and energy-metabolism-related gene responses in juvenile olive flounder under the present experimental conditions. Hepatic *AMPKβ* was the only molecular marker significantly affected by dietary treatment after acute heat exposure, whereas the numerically higher survival observed in some multi-strain groups during the lethal temperature challenge was not statistically significant. Overall, the tested formulations were well tolerated but did not provide clear evidence of improved thermal stress resilience. Future studies incorporating gut microbiome profiling, metabolomic analysis, and alternative dietary or thermal-stress conditions are needed to better evaluate their functional potential in olive flounder aquaculture.

## Figures and Tables

**Table 1:** Formulation and proximate composition of the experimental diets for juvenile olive flounder (g/kg).

Ingredients	CON	AF	BF	CF	ABF	BCF	ACF	ABCF	F
Fish meal (anchovy) ^1^	600	600	600	600	600	600	600	600	600
Starch	50	50	50	50	50	50	50	50	50
Wheat flour	121	121	121	121	121	121	121	121	121
Squid liver powder	50	50	50	50	50	50	50	50	50
Soybean meal	100	100	100	100	100	100	100	100	100
Fish oil	30	30	30	30	30	30	30	30	30
Lecithin	3	3	3	3	3	3	3	3	3
Betaine	3	3	3	3	3	3	3	3	3
Taurine	3	3	3	3	3	3	3	3	3
MCP	3	3	3	3	3	3	3	3	3
Methionine	1	1	1	1	1	1	1	1	1
Lysine	1	1	1	1	1	1	1	1	1
Mineral mix ^2^	12	12	12	12	12	12	12	12	12
Vitamin mix ^3^	12	12	12	12	12	12	12	12	12
Vitamin C	2	2	2	2	2	2	2	2	2
Choline	5	5	5	5	5	5	5	5	5
α-cellulose	5	0	0	0	0	0	0	0	0
FOS	0	5	5	5	5	5	5	5	5
*Bacillus sonorensis*	−	+	−	−	+	−	+	+	−
*Bacillus subtilis*	−	−	+	−	+	+	−	+	−
*Bacillus velezensis*	−	−	−	+	−	+	+	+	−
Total probiotic concentration ^4^	0	1 × 10^7^	1 × 10^7^	1 × 10^7^	1 × 10^7^	1 × 10^7^	1 × 10^7^	1 × 10^7^	0
Proximate analysis (% of dry matter basis)									
Moisture	3.29 ± 1.18								
Crude Protein	57.35 ± 0.82								
Crude Lipid	8.28 ± 0.34								
Crude Ash	14.23 ± 0.65								

Values for ingredients are expressed as g/kg diet, except for probiotic concentration. CON, control diet; AF, *B. sonorensis* + fructo-oligosaccharide (FOS); BF, *B. subtilis* + FOS; CF, *B. velezensis* + FOS; ABF, *B. sonorensis* + *B. subtilis* + FOS; BCF, *B. subtilis* + *B. velezensis* + FOS; ACF, *B. sonorensis* + *B. velezensis* + FOS; ABCF, *B. sonorensis* + *B. subtilis* + *B. velezensis* + FOS; F, FOS-only diet. ^1^ Fish meal and other major feed ingredients were purchased from The Feed Company Limited, Goyang, Republic of Korea. ^2^ Mineral premix was purchased from Duksan Pure Chemicals Company Limited, Ansan, Republic of Korea, and contained the following as g/kg premix: ferrous fumarate, 12.50 g; manganese sulfate, 11.25 g; dried ferrous sulfate, 20.00 g; dried cupric sulfate, 1.25 g; cobaltous sulfate, 0.75 g; zinc sulfate KVP, 13.75 g; calcium iodate, 0.75 g; magnesium sulfate, 80.20 g; and aluminum hydroxide, 0.75 g. ^3^ Vitamin premix was purchased from Duksan Pure Chemicals Company Limited, Ansan, Republic of Korea, and contained the following as mg/kg premix: vitamin A, 1,000,000 IU; vitamin D, 200,000 IU; vitamin E, 10,000 mg; vitamin B1, 2000 mg; vitamin B6, 1500 mg; vitamin B12, 10 mg; vitamin C, 10,000 mg; calcium pantothenate, 5000 mg; nicotinic acid, 4500 mg; biotin, 10 mg; choline chloride, 30,000 mg; and inositol, 5000 mg. ^4^ The final probiotic concentration in each probiotic-containing diet was adjusted to 1 × 10^7^ CFU/g diet. “+” indicates that the specific *Bacillus* strain is included in the diet, and “−” indicates that it is not included.

**Table 2:** List of primers used for gene expression.

Gene Name	Nucleotide Sequences (5′–3′)	Amplicon Size (bp)	Ann. Temp (Tm °C)	Accession Number	References
*β-actin* ^1^	F: GGAATCCACGAGACCACCTACA R: CTGCTTGCTGATCCACATCTGC	264	57.6 58.3	XM_020109620.1	Nie et al. [43]
*AMPKβ* ^2^	F: CCGGGCCATATCATCAGGAC R: TTGTAGCGATGTGTCGCACT	223	56.4 56.5	XM_020103523.1	Nie et al. [44]
*G6pase* ^3^	F: GGGAGCCGCTGGTGTCTAC R: GGCCTTCAGGTACCACTCTTTG	112	58.9 57.0	XM_020109321.1	Yang et al. [45]
*HSP60* ^4^	F: TGACTTCGGGAAAGTCGGTG R: ACGATCTCCAGTGCACGTTT	105	59.3 57.3	XM_020105844.1	NCBI. [46]
*HSP70* ^5^	F: TTCAATGATCTCAGAGGCAAGC R: TTATCTAAGCCTAGGCAATCGC	113	55.4 56.9	XM_020089177.1	Mori et al. [47]
*HSP90α* ^6^	F: GAGCGAGACAAGGAGGTGAG R: CTGGCTTGTCTTCGTCCTTC	100	61.4 59.3	XM_020091873.1	Lee et al. [48]
*HSP90β* ^7^	F: GGAGCTGAACAAGACCAAGC R: CAGATGATCCTCCCAGTCGT	109	59.3 59.3	XM_020097585.1	NCBI. [49]

^1^ *β*-actin: Housekeeping gene; ^2^ 5′ AMP-activated protein kinase subunit beta; ^3^ Glucose-6-phosphatase catalytic subunit; ^4^ Heat shock protein 60; ^5^ Heat shock protein 70; ^6^ Heat shock protein 90α; ^7^ Heat shock protein 90β.

**Table 3:** Growth performance and morphological changes in juvenile olive flounder fed nine experimental diets for nine weeks ^1^.

	Diets ^2^								
	CON	AF	BF	CF	ABF	BCF	ACF	ABCF	F
IBW (g)	7.24 ± 0.21 ^ns^	7.26 ± 0.09	7.28 ± 0.15	7.00 ± 0.03	7.18 ± 0.06	7.35 ± 0.13	7.28 ± 0.15	7.37 ± 0.18	7.32 ± 0.16
FBW (g)	40.7 ± 0.6 ^ns^	41.1 ± 0.6	41.8 ± 0.8	42.5 ± 0.1	39.6 ± 1.5	42.0 ± 1.7	41.2 ± 0.6	40.3 ± 0.62	39.8 ± 1.1
WG (%) ^3^	462 ± 12 ^ns^	467 ± 15	475 ± 22	506 ± 1	452 ± 20	470 ± 14	466 ± 16	448 ± 22	444 ± 25
FE (%) ^4^	126 ± 2 ^ns^	126 ± 2	129 ± 3	135 ± 0	124 ± 5	130 ± 6	128 ± 3	123 ± 3	122 ± 5
SGR (%/day) ^5^	2.78 ± 0.03 ^ns^	2.80 ± 0.04	2.82 ± 0.06	2.91 ± 0	2.75 ± 0.06	2.81 ± 0.04	2.79 ± 0.05	2.74 ± 0.06	2.73 ± 0.07
Survival (%) ^6^	98.1 ± 1.9 ^ns^	90.7 ± 1.9	98.1 ± 1.9	100 ± 3.2	94.4 ± 3.2	94.4 ± 0	96.3 ± 1.9	94.4 ± 0	96.3 ± 3.7
CF ^7^	1.19 ± 0.02 ^ns^	1.20 ± 0.03	1.26 ± 0.04	1.18 ± 0.02	1.18 ± 0.03	1.11 ± 0.04	1.19 ± 0.04	1.16 ± 0.05	1.16 ± 0.06
HSI (%) ^8^	1.54 ± 0.05 ^ns^	1.70 ± 0.07	1.36 ± 0.15	1.52 ± 0.06	1.42 ± 0.05	2.11 ± 0.72	1.38 ± 0.11	1.48 ± 0.11	1.48 ± 0.11
VSI (%) ^9^	3.57 ± 0.25 ^ns^	3.88 ± 0.08	3.72 ± 0.21	3.48 ± 0.07	3.52 ± 0.08	2.86 ± 0.67	3.71 ± 0.13	3.58 ± 0.07	3.58 ± 0.07

^1^ Values are mean ± SEM from three replicate tanks (*n* = 3). Tank means were used for statistical analysis. “ns” indicates *p* > 0.05. ^2^ CON: control; AF: *B. sonorensis* + fructo-oligosaccharide (FOS); BF: *B. subtilis* + FOS; CF: *B. velezensis* + FOS; ABF: *B. sonorensis* + *B. subtilis* + FOS; BCF: *B. subtilis* + *B. velezensis* + FOS; ACF: *B. sonorensis* + *B. velezensis* + FOS; ABCF: *B. sonorensis* + *B. subtilis* + *B. velezensis* + FOS; F: FOS only. ^3^ Weight gain (%) = (final weight − initial weight) × 100/initial weight. ^4^ Feed efficiency (%) = (wet weight gain/dry feed intake) × 100. ^5^ Specific growth rate (%/day) = (ln final weight − ln initial weight) × 100/days. ^6^ Survival rate (%) = (initial number of fish- dead fish) × 100/initial number of fish. ^7^ Condition factor = (wet weight (g)/total length (cm)^3^) × 100. ^8^ Hepatosomatic index (%) = liver weight (g) × 100/body weight (g). ^9^ Viscerosomatic index (%) = Viscera weight (g) × 100/body weight (g).

**Table 4:** Whole-body proximate compositions ^1^ (%, as-is) of juvenile olive flounder fed nine experimental diets for nine weeks ^1^.

	Diets ^2^								
	CON	AF	BF	CF	ABF	BCF	ACF	ABCF	F
Moisture	72.9 ± 0.5 ^ns^	73.7 ± 1.1	73.5 ± 1.1	73.9 ± 0.5	73.7 ± 0.6	72.4 ± 0.3	74.1 ± 1.5	74.6 ± 0.6	73.8 ± 1.0
Crude protein	20.5 ± 0.4 ^ns^	19.9 ± 1.0	20.1 ± 0.8	19.9 ± 0.3	19.9 ± 0.5	20.7 ± 0.9	19.7 ± 1.1	18.9 ± 0.6	19.9 ± 0.9
Crude lipid	3.00 ± 0.14 ^ns^	2.89 ± 0.27	2.76 ± 0.22	2.71 ± 0.29	2.86 ± 0.10	2.77 ± 0.10	2.80 ± 0.32	2.82 ± 0.13	2.83 ± 0.01
Crude ash	4.09 ± 0.10 ^ns^	3.95 ± 0.16	3.95 ± 0.20	3.97 ± 0.01	4.01 ± 0.15	4.12 ± 0.02	3.89 ± 0.17	3.82 ± 0.13	3.59 ± 0.41

^1^ Values are mean ± SEM from three replicate tanks (*n* = 3). Tank means were used for statistical analysis. “ns” indicates *p* > 0.05. ^2^ CON: control; AF: *B. sonorensis* + fructo-oligosaccharide (FOS); BF: *B. subtilis* + FOS; CF: *B. velezensis* + FOS; ABF: *B. sonorensis* + *B. subtilis* + FOS; BCF: *B. subtilis* + *B. velezensis* + FOS; ACF: *B. sonorensis* + *B. velezensis* + FOS; ABCF: *B. sonorensis* + *B. subtilis* + *B. velezensis* + FOS; F: FOS only.

**Table 5:** Plasma metabolites of olive flounder fed nine experimental diets for nine weeks ^1^.

	Diets ^2^								
	CON	AF	BF	CF	ABF	BCF	ACF	ABCF	F
GOT (U/L) ^3^	19.7 ± 0.9 ^ns^	22.0 ± 2.0	18.0 ± 1.0	17.0 ± 1.0	21.7 ± 3.0	18.0 ± 0.6	19.3 ± 1.5	19.3 ± 1.3	19.7 ± 1.7
GPT (U/L) ^4^	19.0 ± 0.0 ^ns^	20.3 ± 2.2	19.0 ± 1.7	17.3 ± 1.5	21.7 ± 2.3	17.7 ± 0.3	18.7 ± 2.3	19.0 ± 1.2	19.7 ± 2.2
GLU (mg/dL) ^5^	19.7 ± 2.9 ^ns^	28.7 ± 5.6	20.7 ± 2.9	24.0 ± 3.2	30.0 ± 15.2	22.7 ± 3.5	25.7 ± 6.3	26.3 ± 8.6	39.0 ± 13.1
TCHO (mg/dL) ^6^	126 ± 13.0 ^ns^	140 ± 5	124 ± 3	116 ± 5	126 ± 11	129 ± 8	124 ± 2	138 ± 8	130 ± 11
TP (g/dL) ^7^	3.13 ± 0.20 ^ns^	3.23 ± 0.07	3.00 ± 0.10	2.97 ± 0.09	3.00 ± 0.31	3.00 ± 0.06	3.03 ± 0.09	3.20 ± 0.15	3.13 ± 0.18
TG (mg/dL) ^8^	255 ± 72.0 ^ns^	272 ± 35	195 ± 8	200 ± 5	210 ± 19	208 ± 22	180 ± 5	235 ± 55	269 ± 55

^1^ Values are mean ± SEM from three replicate tanks (*n* = 3). Tank means were used for statistical analysis. “ns” indicates *p* > 0.05. ^2^ CON: control; AF: *B. sonorensis* + fructo-oligosaccharide (FOS); BF: *B. subtilis* + FOS; CF: *B. velezensis* + FOS; ABF: *B. sonorensis* + *B. subtilis* + FOS; BCF: *B. subtilis* + *B. velezensis* + FOS; ACF: *B. sonorensis* + *B. velezensis* + FOS; ABCF: *B. sonorensis* + *B. subtilis* + *B. velezensis* + FOS; F: FOS only. ^3^ Glutamic oxaloacetic transaminase; ^4^ Glutamic pyruvate transaminases; ^5^ Glucose; ^6^ Total cholesterol; ^7^ Total protein. ^8^ TG: Triglycerides.

**Table 6:** Antioxidant enzymes, immune reaction, and stress-related parameters of juvenile olive flounder fed nine experimental diets for nine weeks ^1^.

	Diets ^2^								
	CON	AF	BF	CF	ABF	BCF	ACF	ABCF	F
GPx (mU/mL) ^3^	253 ± 29 ^ns^	322 ± 27	208 ± 45	216 ± 37	290 ± 55	204 ± 32	197 ± 51	235 ± 10	370 ± 95
SOD (ng/mL) ^4^	553 ± 38 ^ns^	639 ± 70	521 ± 62	640 ± 59	524 ± 38	604 ± 63	538 ± 41	539 ± 40	512 ± 28
IgM (µg/mL) ^5^	1.57 ± 0.09 ^ns^	1.48 ± 0.04	1.51 ± 0.12	1.52 ± 0.11	1.44 ± 0.07	1.55 ± 0.05	1.57 ± 0.05	1.45 ± 0.05	1.42 ± 0.02
LZM (µg/mL) ^6^	7.35 ± 1.16 ^ns^	7.90 ± 0.80	7.16 ± 1.28	9.32 ± 1.25	4.72 ± 2.55	7.02 ± 0.44	7.29 ± 1.00	9.73 ± 1.11	9.09 ± 0.64
HSP70 (pg/mL) ^7^	26.8 ± 6.0 ^ns^	33.5 ± 10.2	23.2 ± 1.0	25.0 ± 4.2	27.4 ± 10.9	19.8 ± 4.8	28.6 ± 8.6	29.3 ± 8.8	24.1 ± 4.1
Cortisol (ng/mL)	3.24 ± 0.3 ^ns^	3.26 ± 0.3	3.55 ± 0.24	4.03 ± 0.38	2.97 ± 0.32	3.02 ± 0.15	3.42 ± 0.08	3.11 ± 0.29	2.70 ± 0.13

^1^ Values are mean ± SEM from three replicate tanks (*n* = 3). Tank means were used for statistical analysis. “ns” indicates *p* > 0.05. ^2^ CON: control; AF: *B. sonorensis* + fructo-oligosaccharide (FOS); BF: *B. subtilis* + FOS; CF: *B. velezensis* + FOS; ABF: *B. sonorensis* + *B. subtilis* + FOS; BCF: *B. subtilis* + *B. velezensis* + FOS; ACF: *B. sonorensis* + *B. velezensis* + FOS; ABCF: *B. sonorensis* + *B. subtilis* + *B. velezensis* + FOS; F: FOS only. ^3^ Glutathione peroxidase; ^4^ Superoxide dismutase; ^5^ Immunoglobulin M; ^6^ Lysozyme; ^7^ Heat shock protein 70.

**Table 7:** Villus length and muscle thickness in the intestine of juvenile olive flounder fed nine experimental diets for nine weeks ^1^.

	Diets ^2^								
	CON	AF	BF	CF	ABF	BCF	ACF	ABCF	F
VH (µm) ^3^	776 ± 18 ^ns^	898 ± 95	862 ± 26	734 ± 246	906 ± 30	792 ± 34	783 ± 84	829 ± 65	793 ± 120
MT (µm) ^4^	59.9 ± 3.5 ^ns^	56.6 ± 4.5	58.2 ± 5.4	56.4 ± 6.0	48.1 ± 8.0	60.5 ± 4.8	63.6 ± 3.7	59.3 ± 5.1	48.1 ± 5.9

^1^ Values are mean ± SEM from three replicate tanks (*n* = 3). Tank means were used for statistical analysis. “ns” indicates *p* > 0.05. ^2^ CON: control; AF: *B. sonorensis* + fructo-oligosaccharide (FOS); BF: *B. subtilis* + FOS; CF: *B. velezensis* + FOS; ABF: *B. sonorensis* + *B. subtilis* + FOS; BCF: *B. subtilis* + *B. velezensis* + FOS; ACF: *B. sonorensis* + *B. velezensis* + FOS; ABCF: *B. sonorensis* + *B. subtilis* + *B. velezensis* + FOS; F: FOS only. ^3^ Villus height; ^4^ Muscular thickness.

**Table 8:** Hematocrit and plasma metabolites of the experimental-diet fed juvenile olive flounder responding to acute temperature exposure ^1^.

	Diets ^2^								
	CON	AF	BF	CF	ABF	BCF	ACF	ABCF	F
Hematocrit (%)	18.3 ± 0.6 ^ns^	18.3 ± 0.7	18.7 ± 0.4	19.2 ± 0.5	17.8 ± 0.4	19.2 ± 0.6	18.8 ± 1.0	19.2 ± 0.5	19.0 ± 0.4
GOT (U/L) ^3^	32.0 ± 7.5 ^ns^	24.0 ± 1.7	37.3 ± 4.9	50.7 ± 23.3	38.3 ± 11.6	26.3 ± 8.9	43.3 ± 17.4	35.0 ± 7.1	21.7 ± 5.7
GPT (U/L) ^4^	25.7 ± 4.1 ^ns^	20.0 ± 2.6	22.7 ± 2.2	21.7 ± 3.2	20.7 ± 2.6	20.7 ± 3.7	27.3 ± 6.9	24.3 ± 5.4	18.7 ± 0.9
GLU (mg/dL) ^5^	47.0 ± 12.5 ^ns^	31.3 ± 5.9	25.3 ± 6.4	24.0 ± 2.1	43.7 ± 10.9	55.3 ± 5.2	41.7 ± 10.2	34.0 ± 10.2	28.7 ± 9.7
TCHO (mg/dL) ^6^	84.0 ± 12.1 ^ns^	100 ± 5	111 ± 6	97.7 ± 4.3	104 ± 2	101 ± 1	101 ± 5	110 ± 5	97.0 ± 3.0
TP (g/dL) ^7^	2.50 ± 0.21 ^ns^	2.73 ± 0.17	2.93 ± 0.17	2.80 ± 0.15	3.00 ± 0.15	2.83 ± 0.09	2.87 ± 0.20	2.93 ± 0.12	3.00 ± 0.0
TG (mg/dL) ^8^	119 ± 15 ^ns^	130 ± 14	158 ± 8	140 ± 9	161 ± 4	124 ± 9	128 ± 7	160 ± 27	147 ± 3
Cortisol (ng/dL)	7.58 ± 0.28 ^ns^	8.56 ± 0.73	10.4 ± 1.70	8.33 ± 0.31	7.00 ± 0.80	7.89 ± 1.43	11.30 ± 2.50	8.61 ± 2.37	9.58 ± 0.69

^1^ Values are mean ± SEM from three replicate tanks (*n* = 3). Tank means were used for statistical analysis. “ns” indicates *p* > 0.05. ^2^ CON: control; AF: *B. sonorensis* + fructo-oligosaccharide (FOS); BF: *B. subtilis* + FOS; CF: *B. velezensis* + FOS; ABF: *B. sonorensis* + *B. subtilis* + FOS; BCF: *B. subtilis* + *B. velezensis* + FOS; ACF: *B. sonorensis* + *B. velezensis* + FOS; ABCF: *B. sonorensis* + *B. subtilis* + *B. velezensis* + FOS; F: FOS only. ^3^ Glutamic oxaloacetic transaminase; ^4^ Glutamic pyruvate transaminase; ^5^ Glucose; ^6^ Total cholesterol; ^7^ Total protein. ^8^ TG: Triglycerides.

**Table 9:** Relative mRNA expression of heat-shock- and energy-metabolism-related genes in juvenile olive flounder after acute heat exposure ^1^.

Diets ^2^	*AMPKβ* ^3^	*G6pase* ^4^	*HSP60* ^5^	*HSP70* ^6^	*HSP90α* ^7^	*HSP90β* ^8^
Liver						
CON	1.01 ± 0.07 ^ab^	1.18 ± 0.50 ^ns^	1.01 ± 0.12 ^ns^	1.04 ± 0.24 ^ns^	1.36 ± 0.75 ^ns^	1.00 ± 0.06 ^ns^
AF	1.20 ± 0.20 ^ab^	1.13 ± 0.44	1.26 ± 0.26	6.89 ± 1.74	0.95 ± 0.23	0.95 ± 0.25
BF	0.98 ± 0.33 ^ab^	1.04 ± 0.17	0.77 ± 0.22	2.76 ± 1.40	0.65 ± 0.06	1.08 ± 0.23
CF	1.23 ± 0.24 ^ab^	1.01 ± 0.33	0.88 ± 0.30	3.05 ± 0.13	1.31 ± 0.73	1.93 ± 1.11
ABF	0.64 ± 0.29 ^b^	1.19 ± 0.24	0.53 ± 0.22	5.17 ± 1.16	2.60 ± 1.93	2.19 ± 1.48
BCF	1.97 ± 0.21 ^a^	0.90 ± 0.33	1.05 ± 0.05	2.68 ± 0.57	0.87 ± 0.49	1.25 ± 0.49
ACF	1.95 ± 0.38 ^a^	1.74 ± 1.20	1.10 ± 0.14	5.70 ± 1.08	1.19 ± 0.57	1.21 ± 0.47
ABCF	1.53 ± 0.12 ^ab^	0.87 ± 0.25	1.26 ± 0.45	6.71 ± 0.00	1.44 ± 0.86	1.60 ± 0.32
F	1.02 ± 0.17 ^ab^	0.99 ± 0.45	0.99 ± 0.28	1.63 ± 0.04	1.15 ± 0.44	1.67 ± 0.77
Brain						
CON	1.32 ± 0.55 ^ns^	1.69 ± 0.94 ^ns^	1.01 ± 0.09 ^ns^	1.05 ± 0.23 ^ns^	1.06 ± 0.24 ^ns^	1.08 ± 0.30 ^ns^
AF	1.51 ± 0.65	2.76 ± 2.14	1.00 ± 0.13	1.08 ± 0.24	1.14 ± 0.47	0.87 ± 0.30
BF	1.25 ± 0.55	2.03 ± 1.37	1.02 ± 0.12	1.27 ± 0.27	1.01 ± 0.19	0.91 ± 0.17
CF	1.26 ± 0.71	1.55 ± 0.67	0.93 ± 0.03	1.04 ± 0.07	1.04 ± 0.18	1.11 ± 0.08
ABF	0.95 ± 0.45	1.24 ± 0.72	0.88 ± 0.10	0.91 ± 0.07	0.91 ± 0.17	0.77 ± 0.07
BCF	1.07 ± 0.46	1.04 ± 0.17	0.93 ± 0.13	0.90 ± 0.11	1.15 ± 0.51	1.01 ± 0.05
ACF	0.92 ± 0.45	1.13 ± 0.52	0.75 ± 0.13	0.91 ± 0.26	0.85 ± 0.12	0.73 ± 0.08
ABCF	1.85 ± 1.30	1.32 ± 0.56	0.85 ± 0.15	1.12 ± 0.35	0.91 ± 0.28	0.91 ± 0.16
F	4.58 ± 3.11	6.37 ± 5.27	0.92 ± 0.08	1.08 ± 0.12	0.98 ± 0.17	0.82 ± 0.14
Gill						
CON	1.03 ± 0.18 ^ns^	1.41 ± 0.84 ^ns^	1.08 ± 0.29 ^ns^	1.32 ± 0.72 ^ns^	1.03 ± 0.20 ^ns^	1.51 ± 0.68 ^ns^
AF	1.04 ± 0.20	1.04 ± 0.52	1.68 ± 0.91	2.48 ± 1.67	1.14 ± 0.59	2.25 ± 1.24
BF	0.78 ± 0.13	1.41 ± 0.76	1.28 ± 0.49	1.95 ± 1.25	1.08 ± 0.33	2.61 ± 0.51
CF	0.66 ± 0.04	3.72 ± 2.60	1.06 ± 0.24	1.83 ± 0.32	1.02 ± 0.09	0.97 ± 0.19
ABF	0.86 ± 0.10	3.84 ± 2.03	1.42 ± 0.35	1.61 ± 0.67	1.95 ± 0.56	3.65 ± 0.68
BCF	0.90 ± 0.14	1.77 ± 0.52	1.93 ± 1.26	3.58 ± 1.75	1.21 ± 0.50	1.49 ± 0.63
ACF	0.98 ± 0.05	3.01 ± 1.42	1.98 ± 0.66	3.51 ± 0.67	1.96 ± 0.63	1.77 ± 0.76
ABCF	0.75 ± 0.09	1.01 ± 0.29	1.02 ± 0.29	2.55 ± 0.66	0.98 ± 0.46	1.13 ± 0.23
F	0.48 ± 0.07	7.61 ± 6.19	1.53 ± 0.60	2.30 ± 0.62	1.12 ± 0.40	2.15 ± 1.27
Kidney						
CON	1.02 ± 0.15 ^ns^	1.03 ± 0.18 ^ns^	1.03 ± 0.20 ^ns^	1.28 ± 0.64 ^ns^	1.07 ± 0.29 ^ns^	1.04 ± 0.18 ^ns^
AF	0.84 ± 0.25	0.90 ± 0.30	0.76 ± 0.16	1.30 ± 0.47	0.89 ± 0.17	0.70 ± 0.18
BF	1.00 ± 0.19	1.48 ± 0.16	0.99 ± 0.05	0.73 ± 0.02	1.10 ± 0.14	1.02 ± 0.13
CF	0.78 ± 0.07	0.77 ± 0.10	0.62 ± 0.22	0.58 ± 0.32	0.79 ± 0.29	0.88 ± 0.09
ABF	1.26 ± 0.24	1.88 ± 0.01	1.06 ± 0.28	1.92 ± 0.72	1.56 ± 0.36	1.44 ± 0.45
BCF	0.97 ± 0.07	1.45 ± 0.69	0.66 ± 0.10	0.58 ± 0.06	1.12 ± 0.43	1.04 ± 0.21
ACF	0.87 ± 0.17	1.22 ± 0.56	0.58 ± 0.10	0.69 ± 0.19	1.09 ± 0.51	0.58 ± 0.15
ABCF	0.88 ± 0.21	1.10 ± 0.58	0.75 ± 0.16	1.42 ± 0.62	0.95 ± 0.24	0.94 ± 0.10
F	0.89 ± 0.23	1.64 ± 0.20	0.64 ± 0.06	0.90 ± 0.29	0.87 ± 0.14	0.76 ± 0.04

^1^ Values are presented as mean ± SEM from three replicate tanks per dietary treatment (*n* = 3). For qPCR analysis, individual fish values within each tank were averaged to obtain one representative tank-level value before statistical analysis. Relative expression levels were calculated using the 2^−ΔΔCt^ method and are expressed as fold changes relative to the control group. Statistical analyses were performed using ΔCt values. Different superscript letters within the same tissue and gene indicate significant differences among dietary treatments based on one-way ANOVA followed by Tukey’s HSD test (*p* < 0.05). “ns” indicates no significant difference. ^2^ CON, control diet; AF, *Bacillus sonorensis* + fructo-oligosaccharide (FOS); BF, *B. subtilis* + FOS; CF, *B. velezensis* + FOS; ABF, *B. sonorensis* + *B. subtilis* + FOS; BCF, *B. subtilis* + *B. velezensis* + FOS; ACF, *B. sonorensis* + *B. velezensis* + FOS; ABCF, *B. sonorensis* + *B. subtilis* + *B. velezensis* + FOS; F, FOS-only diet. ^3^ AMP-activated protein kinase-β; ^4^ Glucose 6-phosphatase; ^5^ Heat shock protein 60; ^6^ Heat shock protein 70. ^7^ Heat shock protein 90α; ^8^ Heat shock protein 90β.

## Data Availability

The data will be available on request.
